# Beyond the Screen: The Impact of Generative Artificial Intelligence (AI) on Patient Learning and the Patient-Physician Relationship

**DOI:** 10.7759/cureus.76825

**Published:** 2025-01-02

**Authors:** Daryl O Traylor, Keith V Kern, Eboni E Anderson, Robert Henderson

**Affiliations:** 1 Public Health, Eastern Washington University, Cheney, USA; 2 Public Health, A.T. Still University (ATSU) College of Graduate Health Studies, Mesa, USA; 3 Basic Sciences, Oceania University of Medicine, San Antonio, USA; 4 Basic Medical Sciences, University of the Incarnate Word School of Osteopathic Medicine, San Antonio, USA; 5 Public Health, A.T. Still University (ATSU) School of Osteopathic Medicine in Arizona, Mesa, USA; 6 Public Health, Eastern Washington University, Spokane, USA

**Keywords:** ethical implications of ai, generative ai, healthcare decision-making, patient education, patient-physician relationship

## Abstract

The rapid advancement of generative artificial intelligence (AI), exemplified by tools like ChatGPT, has transformed the healthcare landscape, particularly in patient education and the patient-physician relationship. While AI in healthcare has traditionally focused on data analysis and predictive analytics, the rise of generative AI has introduced new opportunities and challenges in patient interactions, information dissemination, and the overall dynamics of patient care. This narrative review explores the dual impact of generative AI on healthcare, examining its role in enhancing patients' understanding of medical conditions, promoting self-care, and supporting healthcare decision-making. Additionally, the review considers the potential risks, such as the erosion of trust in the patient-physician relationship and the spread of misinformation, while addressing ethical implications and the future integration into clinical practice. A comprehensive literature search, conducted using databases like PubMed, MEDLINE, Scopus, and Google Scholar, included studies published between 2010 and 2024 that discussed the role of generative AI in patient education, engagement, and the patient-physician relationship. Findings show that generative AI tools significantly enhance patient health literacy by making complex medical information more accessible, personalized, and interactive, thus empowering patients to take a more active role in managing their healthcare. However, risks such as misinformation and the undermining of the patient-physician relationship were also identified, with case studies highlighting both positive and negative outcomes. To fully harness the potential of AI in healthcare, it is essential to integrate these tools thoughtfully, ensuring they complement rather than replace the personalized care provided by physicians. Future research should focus on addressing ethical challenges and optimizing AI's role in clinical practice to maintain trust, communication, and the quality of patient care.

## Introduction and background

Artificial intelligence (AI) has rapidly become an integral part of the healthcare landscape, revolutionizing patient care, diagnostics, and treatment planning [1,2]. Initially, AI's role in healthcare focused on analyzing large datasets to identify patterns, predict outcomes, and support clinical decision-making [3]. For example, AI has been instrumental in advancing predictive analytics, where algorithms can forecast disease progression or identify high-risk patients [4]. Additionally, AI has facilitated the development of personalized medicine by analyzing genetic data and tailoring treatments to individual patients' needs, thereby improving outcomes and reducing adverse effects [5].

However, the scope of AI in healthcare is evolving beyond these traditional applications. A particularly innovative development within this field is the emergence of generative AI tools, such as ChatGPT, which represent a significant shift in how AI interacts with patients and healthcare providers [1,6,7]. Unlike conventional AI systems, which primarily analyze data and make predictions, generative AI is designed to generate human-like text based on extensive training datasets [8]. This capability enables generative AI to engage in meaningful conversations with users, providing information, answering questions, and offering support in a more interactive and personalized manner.

Generative AI tools like ChatGPT can assist patients by providing real-time responses to their inquiries, helping them understand complex medical information, and guiding them through health-related decisions. For example, a patient might use a generative AI tool to learn more about a newly diagnosed condition, understand the implications of lab results, or receive reminders about medication adherence [9]. These tools can also be tailored to provide culturally sensitive information, making healthcare more accessible to diverse populations.

Moreover, generative AI has the potential to bridge gaps in healthcare delivery, especially in areas with limited access to medical professionals [1,10]. By providing immediate, context-specific information, these tools can empower patients to take a more active role in managing their health. The broad dissemination of healthcare knowledge has the potential to reduce disparities in healthcare access and outcomes, particularly for underserved communities.

However, the rise of generative AI in healthcare also presents challenges. There are concerns about the accuracy of information provided by AI, the potential for misuse or over-reliance on these tools, and the ethical implications of AI-generated content [2]. As generative AI becomes more integrated into healthcare, it is vitally important to ensure that these tools are used responsibly, with proper oversight and integration into the broader healthcare system [11].

In summary, generative AI represents a paradigm shift in healthcare by emphasizing human-computer interaction and generating personalized responses tailored to individual patient needs. As these tools continue to evolve, they hold the potential to transform patient education, support self-care, and reshape the patient-physician relationship.

Purpose and scope of the review

The primary focus will be on how patients utilize generative AI to learn about health issues, interpret their health data, and enhance their understanding of personal health management. Additionally, this review article will examine the potential implications of generative AI on the patient-physician relationship, including both the opportunities for enhanced communication and the risks of trust erosion and the addition of confusion in the patient-physician relationship. By investigating these dynamics, the review seeks to provide a comprehensive understanding of the dual-edged nature of generative AI in modern healthcare.

Structure of the paper

The paper is structured to systematically address the key questions surrounding the use of generative AI in patient care. It begins with an exploration of how generative AI is employed by patients for health education and information dissemination. Following this, the paper delves into the use of AI in understanding and interpreting health data, highlighting both its benefits and potential pitfalls. The next section focuses on AI's role in influencing patient self-care and behavior change, considering the ethical implications of such interventions. Finally, the paper discusses the impact of generative AI on the patient-physician relationship, weighing both the threats and enhancements to this critical dynamic. The review concludes with a discussion of the key findings, their implications for clinical practice, and suggestions for future research.

## Review

Methods

A search of the literature was conducted using multiple databases, including PubMed, MEDLINE, Scopus, and Google Scholar, with the aim of identifying relevant studies that discuss the impact of generative AI on patient learning and the patient-physician relationship. The search was conducted between October 7, 2024, and November 10, 2024. The search strategy employed a combination of search terms and MeSH (Medical Subject Headings) terms, including "generative AI," "artificial intelligence," "patient education," "patient-physician relationship," "healthcare," "ChatGPT," "health literacy," "AI in healthcare," "patient engagement," "self-care," and "digital health." Boolean operators (AND, OR) were utilized to refine the search results, and the search was limited to studies published in English.

**Figure 1:**
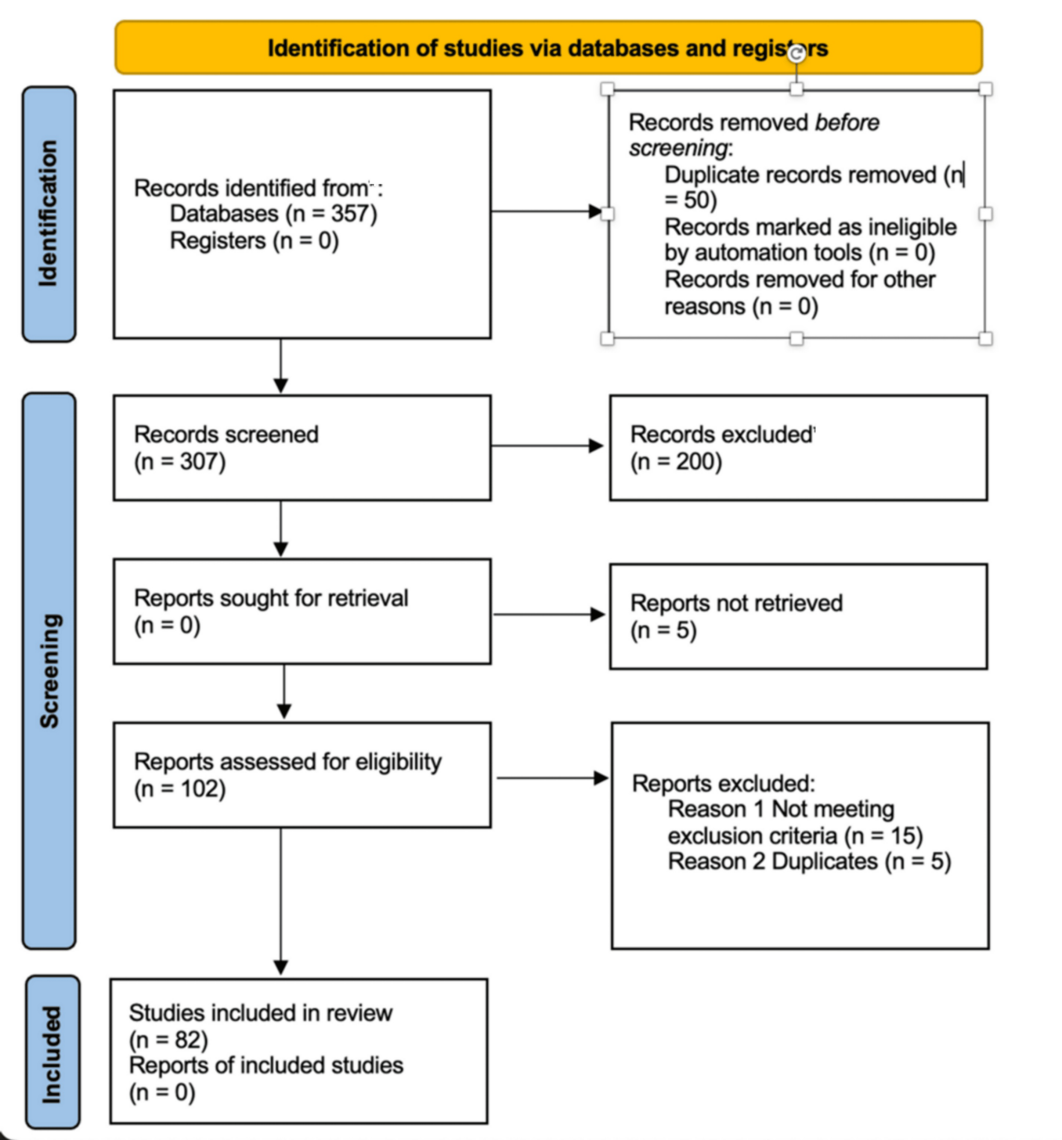
PRISMA 2020 flow diagram for new systematic reviews which included searches of databases and registers only The PRISMA flow diagram illustrates the systematic process used to identify, screen, and include studies for this review. An initial 357 records were identified through database searches. Following the removal of 50 duplicates, 307 records were screened, with 200 excluded due to irrelevance or failure to meet inclusion criteria. Among the 107 reports sought for retrieval, 5 could not be retrieved. Of the 102 reports assessed for eligibility, 20 were excluded - 15 for not meeting inclusion criteria and 5 as duplicates at this stage. Ultimately, 82 studies were included in the review, forming the foundation for the study. PRISMA, Preferred Reporting Items for Systematic Reviews and Meta-Analyses

The inclusion criteria for this narrative review were defined to ensure the relevance and quality of the selected studies. Eligible studies included those published between 2010 and 2024 to cover the most recent developments in generative AI in healthcare. Peer-reviewed articles, reviews, clinical studies, opinion pieces, editorials, and grey literature that discuss the use of generative AI in patient education, patient engagement, or the patient-physician relationship were included. Additionally, the review focused on research that provided data or theoretical analysis on the impact of generative AI tools, such as ChatGPT, on patient outcomes or healthcare dynamics, as well as studies exploring ethical considerations, patient autonomy, and the integration of AI in clinical practice. The process of identifying and including studies is summarized in Figure 1, which presents the PRISMA (Preferred Reporting Items for Systematic Reviews and Meta-Analyses) flow diagram used to document the approach undertaken in this review.

The exclusion criteria were applied to filter out studies that did not align with the review's focus. Articles not available in full text, or those with incomplete data, were excluded, as were studies focusing solely on AI applications outside the scope of patient education, engagement, or the patient-physician relationship (e.g., AI in purely diagnostic or therapeutic roles). Studies published in languages other than English were excluded. Articles that primarily discussed AI technologies unrelated to generative AI, such as machine learning (ML) algorithms for data analysis, were also not considered.

The utilization of generative AI by patients to access health information has grown significantly, driven by the increasing availability and sophistication of AI-powered tools [1,12]. Patients are turning to these tools for several reasons. First, the convenience and immediacy of accessing health information at any time play a crucial role [13]. With generative AI, patients can receive instant answers to their health-related questions without waiting for a scheduled appointment with a healthcare provider. This is particularly appealing to those seeking quick clarifications about symptoms, treatment options, or potential side effects of medications.

Additionally, the anonymity provided by AI tools encourages individuals to inquire about sensitive health issues they may be reluctant to discuss with their physician [14]. For instance, patients might use generative AI to learn more about mental health conditions, sexual health, or stigmatized diseases [15]. The personalized nature of AI-generated responses can be tailored to the user's specific needs and concerns, further enhancing the appeal of these tools.

How generative AI is used for patient education

Generative AI is employed in a variety of ways to educate patients about their health. For example, AI-powered chatbots integrated into health websites or mobile applications can guide patients through a wide range of topics, from understanding a new diagnosis to managing chronic conditions [1,16,17]. These tools can provide explanations of medical terms, suggest lifestyle modifications, and offer tailored advice based on individual health profiles.

Another prominent use case is in medication management. Patients often use generative AI tools to understand how to take their medications properly, including information on dosages, potential interactions, and side effects [18]. AI tools can also help patients interpret lab results or radiology reports by providing explanations that are easier to comprehend than standard medical jargon. However, these applications raise significant safety concerns, particularly regarding the accuracy of the information provided. At this point, it is unclear who could potentially be held liable for misinformation generated by AI. This highlights the need for regulatory frameworks to address these challenges.

Evaluation of the quality of information provided by generative AI

The quality of health information generated by AI is a critical concern, as patients increasingly rely on these tools for making informed decisions about their health [19]. While generative AI has the capability to process and synthesize vast amounts of data from credible sources, the accuracy of the information it provides can vary due to the diverse nature of its training data [20,21]. AI tools like ChatGPT are trained on datasets that include both reliable medical literature and less authoritative sources [21], which can result in the generation of information that may not always align with current medical guidelines or evidence-based practices. These challenges are compounded by the inherent limitations of AI training datasets, which are often derived from publicly available internet sources and may reflect biases, inaccuracies, or outdated information - reflecting the cultural, societal, and historical contexts in which they were created. Furthermore, the underrepresentation of certain demographic groups, languages, and perspectives can lead to gaps in the model’s ability to provide equitable or culturally nuanced responses. The static nature of training data also means that models cannot automatically incorporate new developments, requiring retraining or supplementary tools to remain current. These limitations necessitate critical oversight and ongoing efforts to improve dataset quality and representativeness [22].

To assess the quality of AI-generated health information, it is essential to consider factors such as the algorithms' training data, the frequency of updates, and the mechanisms in place to cross-reference outputs with validated medical sources [19,22]. The static nature of training data means that models cannot automatically incorporate new developments, requiring retraining or supplementary tools to remain current [22]. Moreover, the context in which AI tools are used significantly influences the reliability of the information provided. For example, responses tailored to individual patient queries might inadvertently omit crucial context or oversimplify complex medical concepts [23]. These limitations underscore the need for critical oversight and ongoing efforts to improve the quality, representativeness, and applicability of the datasets used to train AI, ensuring that such tools deliver accurate, equitable, and contextually appropriate health information [19-23].

Potential risks of misinformation

One of the most significant risks associated with generative AI in healthcare is the potential for misinformation [24-26]. Patients who rely solely on AI tools may encounter outdated, incomplete, or inaccurate information, which could lead to poor health outcomes. For example, an AI tool might provide a patient with incorrect guidance on medication usage, leading to adverse effects or ineffective treatment [27]. Furthermore, the nuanced nature of certain medical conditions means that generic AI-generated advice might not be appropriate for all patients, especially those with complex or rare health issues.

The spread of misinformation can also erode trust in both AI tools and healthcare providers, particularly if patients receive conflicting advice from their AI tool and their physician [28-30]. Ensuring the reliability of AI-generated health information is, therefore, crucial for maintaining patient safety and trust.

Role of generative AI in improving patient understanding of health issues

Generative AI has the potential to significantly enhance patient health literacy by making complex medical information more accessible and understandable [30,31]. Generative AI tools can translate dense medical jargon into layman's terms, explain the significance of specific health metrics, and offer visual aids, like diagrams or charts, to help patients grasp intricate concepts [31]. By providing clear, concise explanations, generative AI can empower patients to make more informed decisions about their health and treatment options.

Moreover, these tools can engage patients in an interactive learning process, where they can ask follow-up questions and receive immediate clarification [31]. This interactive capability is particularly beneficial for patients who struggle with understanding their health conditions or the implications of their treatment plans.

Accessibility of generative AI for underserved populations

Generative AI also offers significant accessibility benefits, particularly for underserved populations who may have limited access to healthcare providers [32-34]. For instance, individuals living in remote areas or those facing socioeconomic barriers may use AI tools as a primary source of health information [32,34]. These tools can help bridge the gap by providing accurate, timely information to individuals who might otherwise have difficulty accessing healthcare services.

Additionally, generative AI can be adapted to cater to diverse linguistic and cultural needs, offering translations and culturally relevant information that enhance the accessibility of health education for non-English-speaking or minority populations [32]. By addressing these disparities, generative AI may contribute to a more equitable healthcare landscape, where all patients, regardless of their background, have access to the information they need to manage their health effectively.

How generative AI may help patients interpret and understand their health data

Generative AI may revolutionize the way patients engage with their personal health data, offering tools that help demystify complex medical information [31]. Traditionally, patients have found it challenging to interpret their health records, lab results, and other medical data due to the technical language and intricate details involved. Generative AI addresses this challenge by providing clear, accessible explanations, tailored to the individual’s health context [31,35].

For example, AI tools can analyze a patient's lab results and present the findings in an easy-to-understand format, highlighting key indicators such as cholesterol levels, blood sugar, or blood pressure [31]. Beyond simply translating medical jargon into layman's terms, generative AI can also offer insights into what these results might imply for the patient’s health, suggesting potential areas of concern or improvement [1]. This personalized interpretation enables patients to better understand their health status, ask more informed questions during medical consultations, and take a proactive role in managing their well-being. Additionally, generative AI can integrate data from various sources - such as electronic health records (EHRs), wearable devices, and genetic tests - into a cohesive narrative [1,31,36]. This holistic view allows patients to see connections between different aspects of their health, providing a more comprehensive understanding of their overall health picture [31].

AI’s capability to provide personalized interpretations based on patient data

Generative AI is uniquely equipped to provide personalized health insights by analyzing vast amounts of data specific to an individual [31]. Unlike generic health information, which may not fully account for the nuances of an individual's health history, generative AI can consider a patient’s unique medical records, genetic predispositions, lifestyle factors, and even real-time data from wearable devices to offer tailored advice [31]. For instance, a patient managing diabetes might use a generative AI tool to monitor their blood glucose levels in conjunction with dietary and exercise data [37]. The AI tool can then provide personalized recommendations on how to maintain optimal glucose levels, considering the patient’s specific needs and goals. This level of personalization is a significant advancement over traditional health information sources, which often provide one-size-fits-all advice that may not be relevant to all patients.

Comparison with traditional patient-doctor communication

While generative AI offers valuable personalized insights, it differs fundamentally from traditional patient-doctor communication in several ways. In a typical healthcare setting, physicians interpret patient data and provide recommendations grounded in clinical expertise, while also addressing the patient’s emotional and psychological needs. They contextualize medical information within the broader framework of the patient’s life, incorporating social determinants of health, family history, and unique individual circumstances that AI might not fully comprehend [38,39]. In contrast, generative AI excels at rapidly processing large datasets, identifying patterns that may not be immediately apparent, and delivering consistent, data-driven insights. These capabilities can be particularly beneficial in managing chronic conditions or providing immediate feedback in time-sensitive situations [40].

However, the absence of human empathy and the nuanced understanding that emerges through personal interaction underscores the limitations of AI in healthcare. Patient-physician communication goes beyond the exchange of information; it is the cornerstone of the therapeutic relationship, shaping healthcare quality, patient satisfaction, and outcomes. As healthcare evolves, this interaction remains indispensable, serving as the foundation of compassionate, effective, and personalized care [40,41]. While AI can enhance certain aspects of healthcare delivery, it should be viewed as a complement rather than a replacement for the critical human connection that defines the patient-doctor relationship.

Risks associated with patients relying solely on AI for health data interpretation

While generative AI offers significant benefits in helping patients understand their health data, there are risks associated with relying solely on AI for interpretation [42]. One primary concern is the potential for misinterpretation [42,43]. AI algorithms, despite their sophistication, are not infallible; they can misinterpret data due to limitations in their programming, biases in the datasets they were trained on, or the complexity of an individual’s health condition that exceeds the AI's capabilities [44,45].

For example, an AI tool might incorrectly flag a lab result as abnormal due to a lack of context or a failure to consider individual variations that a human doctor would recognize [44,45]. This could lead to unnecessary anxiety or, conversely, a false sense of security if the AI fails to identify a potential issue [46]. Over-reliance on AI could also result in patients bypassing necessary medical consultations, assuming that the AI's interpretation is sufficient. In one case, a patient assumed their symptoms were related to a recent procedure rather than a more serious condition, such as a stroke. Notably, the way the patient phrased their question to the AI influenced the response. When asked a general question, the AI did not identify stroke as a possibility, but when the question was rephrased to, “Can someone have a stroke after a catheter intervention procedure?” the AI produced a correct response: “Yes, it can" [47]. Fortunately, in this case, there was no adverse outcome, and the patient recovered well with a revision of their medications. This example underscores the importance of nuanced questioning and highlights the potential risks and safety implications of over-relying on AI-generated information.

Studies highlight both the benefits and drawbacks of using generative AI in interpreting health data. Recent advancements in generative AI and ML are transforming the diagnosis and treatment of rare genetic disorders [47]. AI-powered tools can analyze facial images and genetic data to identify potential diagnoses and accurately rank causal genes [48,49]. Other AI systems demonstrate the ability to integrate multiple data sources, including clinical notes and genetic information, to expedite genome interpretation and narrow down candidate diagnoses [50,51]. These technologies enhance diagnostic accuracy by combining various data types, showing great potential in reducing the "diagnostic odyssey" faced by patients with rare diseases, lowering costs, and speeding up case reviews [52,53].

These examples underscore the importance of using generative AI as a supplementary tool, rather than a standalone solution. While AI can enhance patient understanding and engagement with health data, it should always be used in conjunction with professional medical advice to ensure comprehensive and accurate care.

How AI tools encourage or support self-management of chronic conditions, medication adherence, and lifestyle changes

Generative AI tools have become increasingly influential in promoting patient self-care by providing personalized, real-time support for managing chronic conditions, adhering to medication regimens, and making lifestyle changes [18]. For patients with chronic conditions like diabetes, hypertension, or asthma, AI tools can offer tailored advice on monitoring symptoms, adjusting treatment plans, and managing daily routines [54]. These tools may integrate with wearable devices or mobile apps to track health metrics, such as blood glucose levels, blood pressure, or physical activity, offering immediate feedback and suggestions [55]. Some AI models include disclaimers to remind users that the information provided should not replace professional medical advice, reinforcing the importance of consulting healthcare providers for critical decisions.

In terms of medication adherence, generative AI can play a critical role by sending reminders, providing information on the importance of adherence, and offering strategies to overcome barriers. For instance, AI-driven apps can remind patients to take their medication at the correct times, explain potential side effects, and suggest ways to integrate medication into daily routines [18]. This personalized support can help improve adherence rates, particularly in patients who struggle with complex medication regimens.

Generative AI may also support lifestyle changes by offering customized recommendations for diet, exercise, and stress management [18]. These tools can analyze a patient’s current habits and health status and then suggest incremental changes that align with the patient’s goals and preferences. For example, an AI tool might recommend specific dietary adjustments to a patient with high cholesterol or suggest a tailored exercise plan for someone aiming to lose weight [56]. By providing continuous guidance and reinforcement, AI tools can help patients maintain these lifestyle changes over time, ultimately leading to improved health outcomes.

Behavioral change models in AI-driven interventions

**Figure 2:**
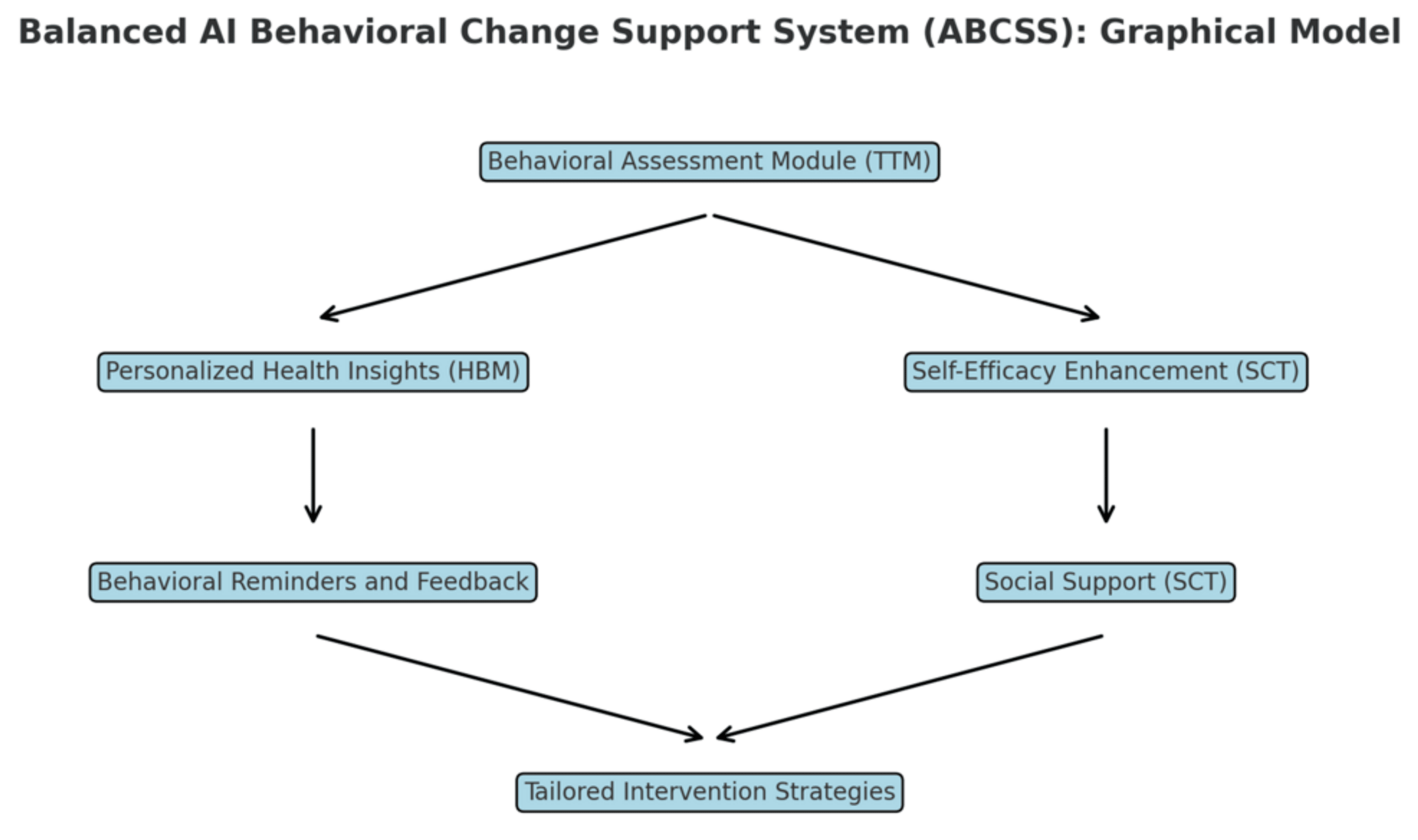
AI Behavioral Change Support System (ABCSS) The ABCSS is a hypothetical model designed to integrate behavioral health theory into AI-driven health interventions. It aims to promote healthy behaviors and support self-management of chronic conditions by incorporating tailored BCTs. The model leverages established frameworks, including the TTM, HBM, and SCT, to provide personalized, dynamic, and contextually relevant support for users. This model would be well-suited for applications such as managing chronic diseases, improving medication adherence, and encouraging lifestyle modifications through generative AI platforms. AI, Artificial Intelligence; ABCSS, AI Behavioral Change Support System; BCT, Behavior Change Technique; TTM, Transtheoretical Model; HBM, Health Belief Model; SCT, Social Cognitive Theory

Generative AI tools incorporate various behavior change techniques (BCTs) to encourage and sustain healthy behaviors. These techniques are grounded in established behavioral science models, such as the Transtheoretical Model (TTM), the Health Belief Model (HBM), and Social Cognitive Theory (SCT) (Figure 2) [57,58]. For instance, AI tools may use goal-setting strategies, where patients are encouraged to set specific, measurable, achievable, relevant, and time-bound (SMART) goals for health improvements [59]. These tools can then track progress and provide positive reinforcement as patients move closer to their goals.

Another common technique is the use of reminders and prompts, which are particularly effective in promoting medication adherence and regular monitoring of health metrics [59]. Generative AI can also employ motivational interviewing principles, where the AI engages patients in reflective dialogues, helping them explore their motivations and barriers to change [60]. This approach fosters a sense of autonomy and self-efficacy, key components in sustaining behavior change.

Moreover, AI tools often utilize feedback loops, where patients receive real-time feedback on their behaviors, allowing them to adjust their actions based on the outcomes [61]. For example, a patient trying to quit smoking might receive daily feedback on their progress, including the health benefits of reduced smoking and tips for managing cravings.

Effectiveness of AI in promoting sustained behavioral changes

The effectiveness of AI-driven interventions in promoting sustained behavioral change has been demonstrated in various studies, particularly in managing chronic diseases and encouraging healthier lifestyles [62,63]. AI tools can provide continuous, personalized support that adapts to the patient's evolving needs, which is crucial for maintaining long-term changes [61]. For example, patients using AI to manage diabetes have reported better glycemic control and higher adherence to recommended dietary and exercise regimens [59].

AI-driven interventions have also been effective in reducing risky behaviors, such as smoking and excessive alcohol consumption. By offering personalized counseling, tracking progress, and providing regular feedback, AI tools can help patients stay motivated and committed to their goals [64]. Additionally, the ability of AI to offer immediate, context-specific advice enhances its effectiveness compared to traditional interventions, which may rely on infrequent interactions with healthcare providers.

However, the success of these interventions often depends on the level of patient engagement and the integration of AI tools into daily life [64]. Patients who actively use these tools and incorporate their recommendations into their routines are more likely to experience sustained behavior change. Therefore, designing AI tools that are user-friendly, engaging, and responsive to individual needs is critical for maximizing their impact.

Ethical considerations

The use of generative AI to influence patient behavior raises several ethical considerations, particularly concerning autonomy, consent, and the potential for unintended consequences. One of the primary ethical concerns is the extent to which AI tools might manipulate patient behavior without their fully informed consent [65]. For instance, AI-driven recommendations could inadvertently pressure patients to make decisions that align with the AI’s programming, but may not reflect the patient’s true preferences or values.

There is also the risk that AI tools could perpetuate biases present in their training data, leading to recommendations that are not equitable across different populations [66]. For example, an AI tool that has been trained primarily on data from a specific demographic group might provide less effective, or even harmful, advice to patients from other backgrounds. Ensuring that AI tools are developed and deployed in a way that respects patient diversity and promotes health equity is crucial.

Moreover, the increasing reliance on AI for health-related decision-making could diminish patient autonomy by creating a dependency on technology [67]. Patients might become less inclined to seek out diverse sources of information or consult with healthcare professionals, instead placing undue trust in AI-generated guidance. This could lead to situations where patients make critical health decisions based on incomplete or inaccurate information.

To address these ethical concerns, it is essential to strike a balance between AI guidance and patient autonomy [66,67]. AI tools should be designed to support, rather than replace, the patient’s decision-making process. This involves ensuring that AI recommendations are presented as options rather than directives, allowing patients to weigh the advice against their own values, preferences, and knowledge [67].

Informed consent is another crucial element in maintaining patient autonomy. Patients should be fully aware of how AI tools work, the limitations of these tools, and the potential risks involved in following AI-generated advice. Transparent communication about the sources of data used by the AI and the rationale behind its recommendations can help patients make more informed choices.

Furthermore, integrating AI tools into a collaborative care model, where AI acts as an adjunct to the healthcare provider’s expertise, can enhance patient autonomy [68]. In this model, patients can use AI to gather information and explore options, but the final decisions are made in consultation with their healthcare provider [68]. This approach ensures that patients receive comprehensive care that considers both the technological insights provided by AI and the personalized, empathetic guidance of a human professional.

In summary, while generative AI has the potential to significantly enhance patient self-care and behavior change, it must be deployed with careful consideration of the ethical implications. By prioritizing transparency, equity, and patient autonomy, AI tools can be valuable allies in promoting healthier behaviors and improving health outcomes.

Potential threats to the patient-physician relationship

Erosion of Trust Due to Conflicting Information Between AI and Physician Advice

One of the most significant potential threats to the patient-physician relationship posed by generative AI is the erosion of trust [69-73]. As patients increasingly turn to AI tools for health information and guidance, they may encounter recommendations that conflict with their physician's advice [72]. These discrepancies can arise from differences in the sources of information, the algorithms used by AI, or the nuances of individual patient cases that AI might not fully appreciate. If patients perceive AI-generated advice as more accessible, immediate, or even more accurate, it may lead to diminished trust in their physician's expertise.

For example, a patient might receive a treatment recommendation from an AI tool that differs from what their physician has suggested, leading to confusion and doubt. This situation can strain the patient-physician relationship, as patients may question the validity of the physician’s advice or feel that their concerns are not being adequately addressed. The risk of conflicting information is particularly pronounced in cases where AI tools are not fully transparent about the data sources or methodologies used to generate their recommendations [74].

Reduced Patient Reliance on Physicians

As AI tools become more sophisticated and widely used, there is also the potential for reduced patient reliance on physicians [75]. Patients may begin to view AI as a more convenient or cost-effective alternative to traditional medical consultations, especially for managing routine health concerns. Over time, this could lead to a shift in the patient’s perception of the physician’s role - from being the primary source of medical guidance to a secondary or confirmatory resource.

This reduced reliance on physicians could have several negative consequences, including the loss of the holistic care that physicians provide, which includes considering the patient’s emotional, psychological, and social needs [75]. Furthermore, the decline in regular patient-physician interactions could lead to missed opportunities for early diagnosis and intervention, as well as a weakening of the trust and rapport that are critical components of effective healthcare [75]. Additionally, the use of AI for diagnosis and treatment recommendations could have significant financial implications, potentially reducing full-time equivalents (FTEs) for physicians. This might lower operational costs but could also result in job displacement and challenges in maintaining adequate workforce expertise.

AI as a Tool for Enhancing Patient Engagement and Shared Decision-Making

While there are potential threats, generative AI also offers opportunities to enhance the patient-physician relationship, particularly in the areas of patient engagement and shared decision-making [76]. AI tools can empower patients by providing them with a better understanding of their health conditions and treatment options, enabling them to engage more actively in discussions with their physicians [77]. When patients come to consultations with a clearer understanding of their health issues, it can lead to more meaningful and productive conversations, where both the patient and the physician collaborate to make informed decisions.

AI can also serve as a valuable adjunct in shared decision-making by presenting patients with evidence-based options and helping them weigh the potential benefits and risks of different treatments [78]. This can enhance the quality of care by ensuring that decisions are made based on a comprehensive understanding of the available data, aligned with the patient’s values and preferences.

Strategies for Integrating AI Into Clinical Practice to Support the Patient-Physician Relationship

To maximize the benefits of AI while mitigating potential challenges, it is essential to develop strategies for effectively integrating AI into clinical practice. One approach is to position AI as a complementary tool that supports, rather than replaces, the physician’s role [79]. For example, physicians can use AI-generated insights as a starting point for discussions with patients, ensuring that the final decisions are made collaboratively.

Another strategy is to enhance the transparency of AI tools, helping patients understand how AI generates its recommendations and the limitations of these tools [80]. By fostering an open dialogue about the role of AI in healthcare, physicians can build trust and ensure that patients view AI as an aid to their care rather than a replacement for human expertise.

Training physicians to effectively incorporate AI into their practice is also crucial [81]. This includes understanding how to interpret AI-generated data, how to communicate these insights to patients, and how to address any concerns that arise from AI-driven recommendations [82]. By equipping physicians with the skills to navigate the evolving landscape of AI in healthcare, the patient-physician relationship can be strengthened rather than undermined.

AI Bias and Its Intersections With the Patient-Physician Relationship

Generative AI systems, despite their potential for innovation, have been shown to perpetuate biases inherent in their training data, raising significant concerns for the patient-physician relationship [77]. AI bias often stems from the use of historical data that reflects societal inequalities, particularly regarding race, gender, and socioeconomic status [81,82]. When applied in medical contexts, these biases can lead to skewed decision-making, misdiagnoses, or unequal treatment recommendations [77]. This not only undermines the effectiveness of AI as a tool but also poses a threat to the trust integral to the patient-physician relationship. For instance, if AI systems consistently provide suboptimal care for marginalized groups, it may erode confidence in medical interventions and exacerbate health disparities [77]. Patients may feel alienated or mistrustful of their physicians, particularly if they perceive that clinical decisions are being overly influenced by flawed algorithms rather than personalized, compassionate care. Therefore, addressing AI bias is crucial not only for ensuring equitable healthcare outcomes, but also for preserving the integrity and trust essential to patient-physician interactions.

In summary, while generative AI presents certain risks to the patient-physician relationship, it also offers significant opportunities to enhance patient engagement, improve communication, and support shared decision-making. By carefully managing the integration of AI into clinical practice, these tools can be harnessed to strengthen the patient-physician dynamic, ultimately leading to better health outcomes.

Discussion

Generative AI has emerged as a powerful tool in healthcare, significantly impacting patient learning, understanding of health data, and self-care practices. Patients increasingly use AI tools to access and understand health information, which has democratized access to knowledge and empowered patients to take a more active role in managing their health. These tools simplify complex medical concepts, provide personalized health insights, and support self-management of chronic conditions, medication adherence, and lifestyle changes.

However, the integration of AI into patient care is not without challenges. While AI may enhance patient engagement and self-care, it also raises concerns about the accuracy and reliability of the information provided, as well as the potential for patients to misinterpret or overly rely on AI-generated advice. A significant concern is that patients might substitute AI for the need for physical examinations or direct physician contact, potentially overlooking the importance of in-person assessments for accurate diagnoses and holistic care. While this could reduce out-of-pocket costs for some patients, it also risks delaying necessary medical interventions, thereby increasing long-term healthcare costs and jeopardizing patient outcomes. The dual nature of AI's influence is particularly evident in its potential impact on the patient-physician relationship. On one hand, AI might facilitate more informed and collaborative decision-making between patients and physicians; on the other hand, it can also create tensions when AI-generated information conflicts with physician advice, potentially eroding trust and reducing reliance on physicians.

The impact of generative AI on the patient-physician relationship is complex and multifaceted. AI has the potential to enhance this relationship by providing patients with the tools they need to engage more deeply in their care, fostering a more collaborative dynamic. However, there is also the risk that AI could undermine the relationship by creating conflicting sources of authority, leading patients to question or even disregard their physician's advice. The challenge lies in balancing the benefits of AI with the need to maintain the trust, communication, and personalized care that are central to the patient-physician relationship.

Implications for Clinical Practice

Recommendations for physicians in navigating the use of AI by patients: As AI becomes more integrated into patient care, physicians will need to adapt to a new landscape where patients come to consultations with AI-generated information. To navigate this shift, physicians should prioritize clear and open communication, acknowledging the role of AI while also reinforcing their expertise and the value of personalized care. Physicians should educate patients on the strengths and limitations of AI tools, guiding them on how to use these resources effectively, without over-reliance.

Physicians may also need to incorporate AI into their own practice, using AI-generated insights as a supplementary tool to enhance patient care. By embracing AI as a partner in the healthcare process, physicians can help ensure that these technologies are used to support rather than disrupt the patient-physician relationship.

Future role of AI and its integration into healthcare systems

Looking forward, the role of AI in patient care is likely to expand, with AI becoming an integral part of healthcare systems. The future of AI in healthcare will involve deeper integration into clinical workflows, where AI tools assist with everything from diagnostics to treatment planning and patient follow-up. For AI to be successfully integrated, healthcare systems must focus on developing robust frameworks for AI use, ensuring that these tools are evidence-based, transparent, and aligned with clinical best practices.

The future role of AI will also depend on ongoing education and training for both healthcare professionals and patients. As AI technologies continue to evolve, it will be essential for healthcare providers to stay informed about the latest developments and cultivate digital literacy among patients, enabling them to use AI tools safely and effectively.

Areas for future research

Identifying Gaps in the Current Literature

While there is a growing body of research on the role of AI in healthcare, significant gaps remain. One key area that requires further exploration is the long-term impact of AI on patient outcomes, particularly in diverse populations. Current studies often focus on short-term effects or specific use cases, leaving a need for comprehensive, longitudinal research that examines how AI influences health over time.

Another critical gap in the literature is the impact of AI on health disparities and its intersection with systemic racism. While AI has the potential to improve access to care and enhance health outcomes, there is a significant risk that it could exacerbate existing inequities if not designed and implemented with equity in mind. Algorithmic biases, often rooted in underrepresented or misrepresented data, can perpetuate systemic disparities by leading to inequitable treatment recommendations, misdiagnoses, or the exclusion of marginalized groups from the benefits of AI-driven advancements. Future research should explore how AI affects different demographic groups - particularly in terms of access, quality of care, and health outcomes - to ensure that these technologies promote health equity rather than reinforce systemic inequities.

Suggestions for future studies on AI’s impact on healthcare

Future studies should focus on several key areas to deepen our understanding of AI's impact on healthcare. First, research should investigate the effectiveness of AI in supporting chronic disease management and behavioral change over the long term, exploring how AI tools can be optimized to sustain patient engagement and improve outcomes.

Second, there is a need for studies that examine the ethical implications of AI in healthcare, particularly in terms of patient autonomy, consent, and the potential for bias. Researchers should develop frameworks for assessing the ethical use of AI and propose strategies for mitigating risks.

Finally, future research should explore the integration of AI into clinical practice, examining how AI tools can be used to complement the physician’s role without undermining the patient-physician relationship. This includes studying the most effective ways to train healthcare professionals in using AI, and assessing the impact of AI on healthcare delivery and patient satisfaction.

## Conclusions

In conclusion, generative AI presents transformative opportunities for healthcare, but its successful integration into patient care requires thoughtful implementation, ongoing research, and a commitment to addressing the ethical and practical challenges it poses. Healthcare professionals must actively guide patients in using AI as a supplemental tool rather than a substitute for expert medical care, to preserve the integrity of the patient-physician relationship. By fostering transparent communication, reinforcing the centrality of trust, empathy, and personalized care, and addressing issues such as bias and equity, the healthcare community can harness AI's potential to enhance patient outcomes. Through a balanced approach that safeguards the human connection fundamental to healing, AI can become a powerful ally in advancing the future of medicine.
